# Antibody-drug conjugates: recent advances in conjugation and linker chemistries

**DOI:** 10.1007/s13238-016-0323-0

**Published:** 2016-10-14

**Authors:** Kyoji Tsuchikama, Zhiqiang An

**Affiliations:** Texas Therapeutics Institute, The Brown Foundation Institute of Molecular Medicine, The University of Texas Health Science Center at Houston, Houston, TX 77054 USA

**Keywords:** antibody-drug conjugates, cancer, chemotherapy, conjugation, linker, site-specific conjugation

## Abstract

The antibody-drug conjugate (ADC), a humanized or human monoclonal antibody conjugated with highly cytotoxic small molecules (payloads) through chemical linkers, is a novel therapeutic format and has great potential to make a paradigm shift in cancer chemotherapy. This new antibody-based molecular platform enables selective delivery of a potent cytotoxic payload to target cancer cells, resulting in improved efficacy, reduced systemic toxicity, and preferable pharmacokinetics (PK)/pharmacodynamics (PD) and biodistribution compared to traditional chemotherapy. Boosted by the successes of FDA-approved Adcetris^®^ and Kadcyla^®^, this drug class has been rapidly growing along with about 60 ADCs currently in clinical trials. In this article, we briefly review molecular aspects of each component (the antibody, payload, and linker) of ADCs, and then mainly discuss traditional and new technologies of the conjugation and linker chemistries for successful construction of clinically effective ADCs. Current efforts in the conjugation and linker chemistries will provide greater insights into molecular design and strategies for clinically effective ADCs from medicinal chemistry and pharmacology standpoints. The development of site-specific conjugation methodologies for constructing homogeneous ADCs is an especially promising path to improving ADC design, which will open the way for novel cancer therapeutics.

## INTRODUCTION

Over the past half century, cancer management has improved significantly along with the advancement of chemotherapy (DeVita and Chu, 2008). Chemotherapy using cytotoxic agents is a major treatment option, in addition to surgical removal, radiation, targeted therapies using small molecules or monoclonal antibodies (An, 2010), and, more recently, immunotherapy. Chemotherapy has been refined through screening and development of small molecules that can cause cell death selectively to cancer cells through inhibiting microtubule function, DNA synthesis, or protein function. Although chemotherapy has seen great success in treatment of cancer, especially leukemia, difficult issues remain, such as the development of resistance mechanisms. Severe adverse effects derived from off-target cytotoxicity may worsen a patient’s quality of life, contributing to discontinuation of medication. This fact has discouraged clinicians and medicinal chemists from pursuing more highly potent cytotoxic agents for cancer treatment. In this context, the use of highly cytotoxic agents conjugated with cell-targeting molecules emerged as a potential clinical strategy. In particular, antibody-drug conjugates (ADCs), humanized or human monoclonal antibodies conjugated with cytotoxic small molecules through chemical linkers, could potentially make a fundamental change in the way cancer chemotherapy is designed and administered (Chari et al., 2014; Perez et al., 2014; Bouchard et al., 2014; Jain et al., 2015; McCombs and Owen, 2015; Chudasama et al., 2016; Diamantis and Banerji, 2016). This platform enables targeting cancer cells and selective delivery of highly cytotoxic drugs, resulting in a broad therapeutic window. Indeed, successful clinical outcomes using ADCs have inspired scientists in the biomedical research community to further advance this new platform towards next-generation cancer therapeutics. In this article, we review molecular aspects of ADCs, successful ADCs currently used in clinical application, and recent progress in the conjugation and linker technologies for successful construction of ADCs.

## BRIEF HISTORY OF ADC

The concept of selective delivery of toxic agents to target cells causing disease was originally proposed in 1913 by German physician and scientist Paul Ehrlich (Ehrlich, 1913). Forty five years later, his concept of targeted therapy was first demonstrated in the form of an ADC, methotrexate conjugated to a leukemia cell-targeting antibody (Mathe et al., 1958). In early studies, polyclonal antibodies were the main targeting agents. The first ADC human clinical trial was conducted using an anti-carcinoembryonic antigen antibody-vindesine conjugate in 1983 (Ford et al., 1983), and a promising outcome was reported. Technological advancements in antibody engineering, including production of humanized antibodies, boosted studies on ADC. The first-generation ADCs consisting of chimeric or humanized antibodies, were tested in the 1990s. Finally, further significant efforts towards practical therapeutics led to FDA-approved ADCs: gemtuzumab ozogamicin (Mylotarg^®^) in 2000 for CD33-positive acute myelogenous leukemia (Sievers et al., 2001), brentuximab vedotin (Adcetris^®^) in 2011 for CD30-positive relapsed or refractory Hodgkin’s lymphoma and systemic anaplastic large cell lymphoma (Younes et al., 2010), and trastuzumab emtansine (Kadcyla^®^) in 2013 for HER2-positive breast cancer (LoRusso et al., 2011; Verma et al., 2012). However, Mylotarg^®^ was withdrawn from the market in 2010 due to a lack of clinical benefit and high fatal toxicity rate compared to the standard chemotherapy (ten Cate et al., 2009). In spite of this setback, ADC technologies have been rapidly evolving and about 60 ADCs are currently in clinical trials (Diamantis and Banerji, 2016). In addition to immunotherapy with checkpoint inhibitors (Postow et al., 2015), this emerging molecular platform for chemotherapy is predicted to significantly increase its share of the market as one of the most effective anti-cancer therapeutics in the near future (Mullard, 2013).

## STRUCTURE AND MECHANISM OF ACTION OF ADC

**Figure 1:**
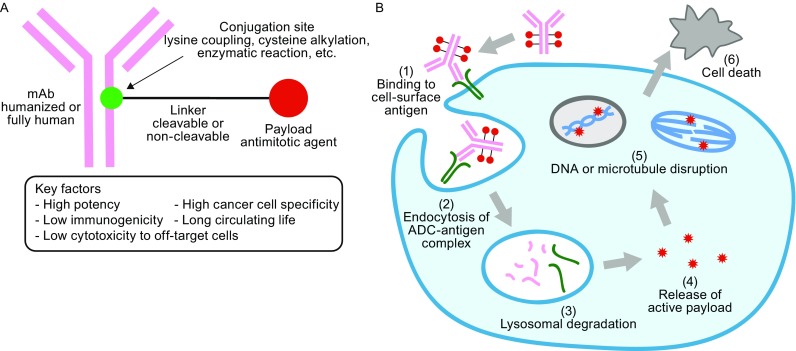
**Structure and mechanism of action of ADC**. (A) A general structure of an ADC containing a humanized/human monoclonal antibody (mAb), a cleavable/non-cleavable chemical linker, and a cytotoxic payload. The linker is covalently linked to the mAb at the conjugation site. (B) A general mechanism of action of ADCs. The ADC binds to its target cell-surface antigen receptor (Step 1) to form an ADC-antigen complex, leading to endocytosis of the complex (Step 2). The internalized complex undergoes lysosomal processing (Step 3) and the cytotoxic payload is released inside the cell (Step 4). The released payload binds to its target (Step 5), leading to cell death (Step 6)

**Figure 2:**
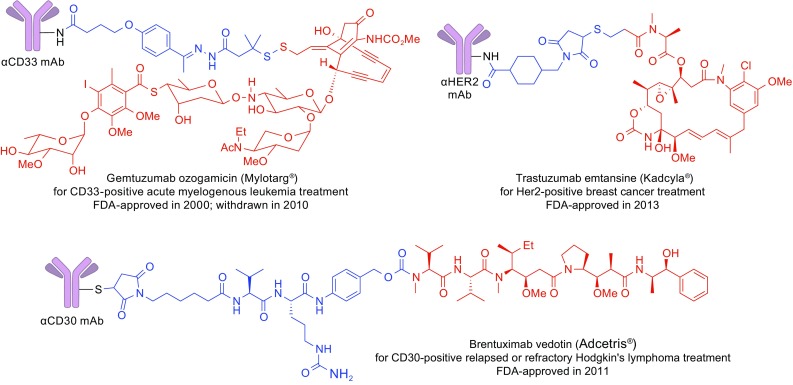
**Structures of FDA-approved ADCs: Mylotarg** ^**®**^ **, Adcetris** ^**®**^ **, and Kadcyla** ^**®**^ **(blue: linker, red: payload)**

ADCs comprise monoclonal antibodies and cytotoxic agents (payloads) covalently conjugated through chemical linkers (Fig. 1A). In modern research and development of ADCs, humanized or fully human monoclonal antibodies (hmAbs) are the first choice of delivery platform to secure high cell target specificity, long circulating half life in human bloodstream (up to three weeks in the case of immunoglobulin G (IgG)), and minimal immunogenicity. A general mechanism of action of ADCs is depicted in Fig. 1B. After ADC molecules are administered into the blood stream, the antibody component of the ADC recognizes and binds to cell-surface antigens that are highly expressed in target cancer cells. Upon internalization of the ADC-antigen complex through endocytosis, the complex is processed within lysosomes, which releases the cytotoxic payload (antimitotic agents in general) in a bioactive form inside the cell. The released payload disrupts DNA strands or microtubules, or exerts topoisomerase or RNA polymerase inhibition, leading to cell death. Cytotoxic chemical agents that have high potency to cancer cells but low off-target cytotoxicity are generally used as payload. Chemical structures of Mylotarg^®^, Adcetris^®^, and Kadcyla^®^ are depicted in Fig. 2.

## CHOICE OF ANTIGEN AND PAYLOAD

Given the mechanism of action, the ideal antibody needs to have sufficient antigen affinity and specificity. However, antibodies with extremely high antigen affinity are known to lead to reduced efficiency of solid tumor penetration (Rudnick et. al., 2011). Thus, ADCs with high antigen affinity do not necessarily lead to high clinical efficacy. In addition, cell-surface antigens must be predominately expressed on target cells with minimal expression on healthy cells to achieve effective drug delivery and selective killing of tumor cells, which determines the therapeutic window. In this context, one may think tumor antigen density directly correlates to efficacy of ADCs. However, several studies suggest that the correlation between antigen density and ADC efficacy depends on the type of cancer cells (Polson et al., 2011; Kung Sutherland et al., 2013) due to varying internalization rate of each antigen after formation of a complex with an ADC molecule. While important to select a cancer cell-specific antigen, the prediction of total efficacy of ADCs based on the antigen expression level remains elusive (Damelin et. al., 2015).

Another important consideration is the limited number of payload molecules that can be efficiently delivered into target cells. Only 1.56% of administered drug molecules can enter target cells if the efficiency of each step in the ADC mechanism is assumed to be 50% (biodistribution, binding to antigen, internalization, release of payload, intracellular stability of payload, and payload binding to target) (Teicher and Chari, 2011). Indeed, the actual uptake is estimated to be much lower than this assumption (<0.01% injected dose per gram of tumor) (Sedlacek et al., 1992). Thus, to maximize treatment efficacy using ADCs, cytotoxic potency of payload is required to be high enough to effectively eradicate target cells, ideally in the picomolar range. While important to select highly potent toxic agents as payload, ideal agents have inherent selectivity for target cancer cells. Certain types of noncancerous cells may be capable of internalizing ADCs through nonspecific pinocytosis or fragment crystallizable (Fc) region receptor-mediated endocytosis (Lencer and Blumberg, 2005). Furthermore, payload may be released upon degradation into circulating blood. Thus, payloads have primarily been selected based on the above-mentioned consideration; antimitotic agents, which are generally less toxic to noncancerous cells than to cancerous cells, are payloads that have been mainly used in the FDA-approved ADCs and ADCs in clinical trials. In addition to calicheamicins (used in Mylotarg^®^), auristatins (used in Adcetris^®^), and maytansinoids (used in Kadcyla^®^), new classes of highly potent antimitotic compounds have also been explored for ADC payloads: duocarmycins, pyrrolobenzodiazepine dimers (PBDs), amanitins, and tubulysin analogs are such examples (Chari et al., 2014; Perez et al., 2014).

## THE CONJUGATION AND LINKER CHEMISTRIES FOR ADC

Though it is important to select optimal target-specific antibodies and potent payloads based on type of cancer cells, the conjugation and linker chemistries are also crucial components for successful construction of an ADC and the major topics of this review. The linker moiety covalently tethers the antibody and payload components. Its molecular design and properties are critical determinant factors for ADC efficacy in terms of pharmacokinetics (PK)/pharmacodynamics (PD) and therapeutic window. To maximize these parameters, various types of linkers have been developed and evaluated *in vitro* and *in vivo*. Several criteria must be met for successful ADC construction. (1) The linker needs to possess sufficient stability in plasma so that ADC molecules can circulate in the bloodstream and localize to the tumor site without premature cleavage. Instability of the linker causes premature liberation of the toxic payload and undesired damage to non-target healthy cells, which can lead to systemic toxicity and adverse effects. However, a clinical study revealed reverse correlation between linker stability of maytansinoid-based ADCs and adverse toxicity (Drake and Rabuka, 2015). Therefore, it is important to identify ADC linkers with optimal linker stability for each combination of antigen, target tumor type, and payload. (2) At the same time, the linker needs to possess the ability to be rapidly cleaved and to release free and toxic payload once the ADC is internalized into the target tumor cell. (3) Another property to be considered in the linker design is hydrophobicity. Hydrophobic linkers coupled with hydrophobic payloads often promote aggregation of ADC molecules. For example, King and co-workers observed non-covalent dimerization of the monoclonal antibody BR96 conjugated with doxorubicin through a multi-loading, hydrophobic dipeptide linker (King et al., 2002). Such molecules are unfavorable in the pursuit of therapeutically useful ADCs; aggregated proteins tend to be rapidly sequestered in the liver and cleared by the reticuloendothelial system, resulting in hepatotoxicity (Finbloom et al., 1980). In addition, aggregated proteins are likely to function as immunogenic substances, provoking undesired immune response during circulation in bloodstream. This problem can be overcome by employing hydrophilic linkers containing negatively charged sulfonate groups (Zhao et al., 2011), polyethylene glycol (PEG) groups (Lyon et al., 2015), or pyrophosphate diester groups (Kern et al., 2016).

Based on the above-mentioned criteria, tremendous effort has been put toward developing conjugation methods and ADC linker structures. Chemical conjugation and enzymatic conjugation are two methods for tethering the antibody and payload components that are currently in use. Linker structure is categorized into two major classes based on the payload release mechanism: cleavable or non-cleavable linker. Herein, we review modern conjugation methods and ADC linker technologies in detail.

### Chemical conjugation

In ADC chemical conjugation, accessible amino acid residues on the surface of the antibody undergo a controlled reaction with a reaction handle installed on the linker. Depending on the chemical conjugation method selected, this process affords a mixture of ADC species with variable Drug-Antibody Ratios (DARs) and tethering sites. In general, a broad distribution of DAR can lead to reduced efficacy, and thus the distribution needs to be tightly controlled. High DAR can increase not only potency but also the risk of aggregation, clearance rate, and premature release of the toxic payload during circulation. This risk can be reduced by employing hydrophilic, sufficiently stable linkers. Overall, it is crucial to identify an optimal DAR value with a controlled distribution for each ADC that can maximize the balance of efficacy, tolerability, and cytotoxicity profiles.

#### Lysine amide coupling

**Figure 3:**
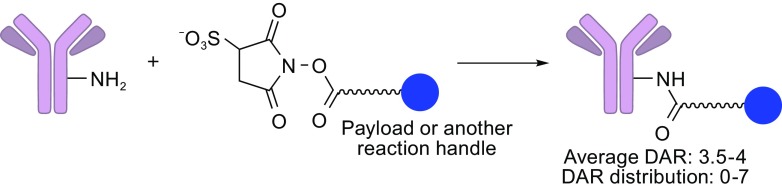
**Lysine amide coupling**. An activated carboxylic acid moiety reacts with a lysine residue, which results in amide bond linkage between mAb and the payload. Optimized conjugation conditions give an average drug-to-antibody ratio (DAR) value of 3.5–4 with distribution between 0–7

Amide coupling is a major ADC conjugation method connecting a payload and solvent accessible lysine residues on the antibody using linkers containing activated carboxylic acid esters (Fig. 3). Amide coupling of an amine and an activated carboxylic acid is one of the most reliable, high-yielding chemical conversions in organic synthesis. However, there are about 80 lysine residues on a typical antibody and about 10 residues are chemically accessible (Chari, 2008). Thus, this conjugation modality often gives multiple ADC species with variable DARs and conjugation sites. In the case of a maytansinoid-type ADC, the average DAR was 3.5–4 with distribution between 0–7 (Lazar et al., 2005). As described above, DAR and its distribution critically impact PK/PD and cytotoxicity of ADCs. Furthermore, some lysine residues that are critical in antibody-antigen interactions may be modified, resulting in reduced binding affinity. As such, heterogeneous mixtures of ADCs constructed using this conjugation method could potentially lead to a poor therapeutic index. While achievable as seen in the FDA-approved Kadcyla^®^ and clinically tested ADCs, the lysine-based conjugation requires effort to develop reproducible manufacturing processes ensuring controlled DAR and distribution within a target range (typically 3–4 as a major species).

#### Cysteine coupling

**Figure 4:**
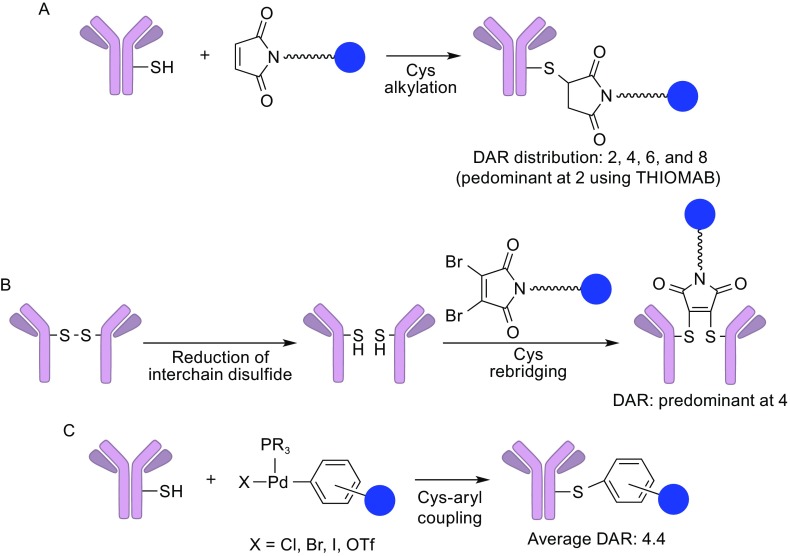
**Cysteine coupling**. (A) Maleimide alkylation. A maleimide moiety reacts with a reduced cysteine residue of a mAb (distribution of DAR: 2, 4, 6, and 8 or predominant at 2 with THIOMAB technology). (B) Rebridging of interchain disulfide bonds. The dibromo (or disulfonate) reagent reacts with the reduced interchain disulfides to provide rebridged mAbs (DAR: predominant at 4). (C) Cysteine arylation using palladium complexes. Aryl-palladium complex reagents undergo aryl-thiol coupling, which affords mAbs containing arylcysteines (average DAR: 4.4)

Cysteine-based conjugation methods rely on a specific reaction between cysteine residues of the antibody and a thiol-reactive functional group installed on the payload (Fig. 4A). In general, antibodies do not possess free thiols, and all cysteine residues form disulfide bonds. In human IgG1, which is most commonly used in modern ADCs, there are 4 interchain and 12 intrachain disulfide bonds. The 4 interchain disulfides, which are generally not critical for structural stability of IgG1, can be selectively reduced under mild conditions to give 2, 4, 6, or 8 free thiols while keeping the 12 intrachain disulfides intact. Due to the limited number of conjugation sites and the distinct reactivity of the thiol group, cysteine-based conjugation is superior to lysine-based conjugation in terms of controlled DAR and heterogeneity. As is the case with the lysine-based conjugation, this conjugation method was a major choice for ADC construction and used for Adcetris^®^ and many other ADCs in clinical trials. However, this modality still has room for improvement to achieve better DAR and heterogeneity control: the above-mentioned simple cysteine conjugation can give a DAR distribution raging from 0 and 8. Junutula and co-workers introduced two new cysteine residues (one per heavy chain) for selective antibody attachment (Junutula et al., 2008). This engineered cysteine technology, THIOMAB, enables generation of highly homogeneous ADCs with a DAR of 2 (>90% homogeneity). ADCs constructed using this technology have shown quite encouraging results (high efficacy and therapeutic window) in *in vivo* studies (Junutula et al., 2008). Cysteine rebridging is another strategy that was recently developed to better control DAR and heterogeneity of ADCs. Dibromomaleimide (Behrens et al., 2015; Bryden et al., 2014), dibromopyridazinediones (Maruani et al., 2015), and a 1,3-bis(*p*-toluenesulfonyl)propane-based core (Bryant et al., 2015) can accept two reduced cysteines derived from interchain disulfide bonds to afford a rebridged antibody (Fig. 4B). These site-specific conjugations theoretically provide many advantages in terms of structural stability, homogeneity, and well-controlled DAR (predominant at 4 in the case of dibromomaleimide) (Behrens et al., 2015). Coupled with selection of proper linker structure and payload, this method can potentially lead to ADCs with enhanced PK/PD and therapeutic efficacy.

Recently, Buchwald and co-workers developed a rapid, highly selective cysteine conjugation using aryl palladium complexes (Vinogradova et al., 2015) (Fig. 4C). The aryl palladium reagents are readily prepared by mixing active palladium-phosphine complexes and various aryl halides. The resulting complexes undergo a thiol arylation with reduced cysteine residues of the antibody in a rapid and selective manner. They demonstrated the potential of this new method in direct conjugation with trastuzumab and a palladium complex of vandetanib (a kinase inhibitor), which gave a linker-free ADC with a DAR of 4.4. Although the obtained ADC lacked a linker, it retained binding affinity to recombinant HER2 (K_d_ = 0.1–0.5 nmol/L) comparable to that of the parent trastuzumab. Another advantage of this method is that the resulting aryl-cysteine conjugates are stable towards acids, bases, oxidants, and externally added thiols. While unique and intriguing, this method needs substantial modification or improvement of several critical factors for the future clinical application (toxicity of palladium, workup strategies for complete removal of palladium, cost for palladium complexes, DAR control, etc.). Despite current limitations, further work will provide researchers with profound insights into cysteine-based conjugation chemistry and rational design of reagents for preparing ADCs that could have not been constructed with traditional methods.

#### Non-natural amino acid incorporation by genetic engineering

**Figure 5:**
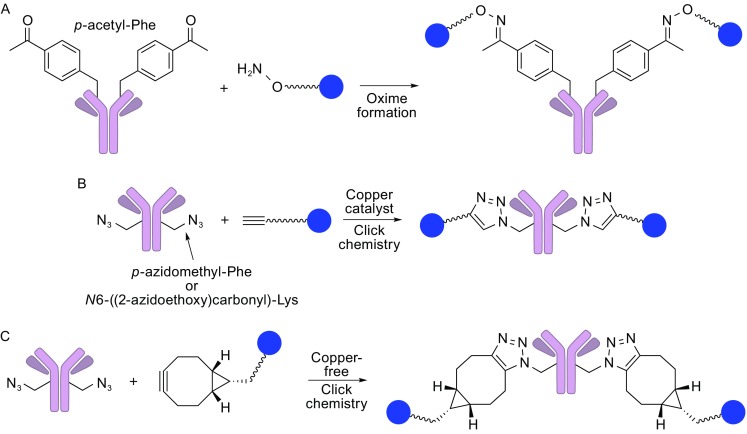
**Non-natural amino acid incorporation by genetic engineering into mAbs and subsequent chemical conjugation**. (A) Oxime ligation. (B) Copper-catalyzed or (C) strain-promoted (copper-free) azide-alkyne cyclization. The site-specific conjugation method gives a defined DAR value depending on the number of non-natural amino acid residues that are genetically incorporated

Installation of non-natural amino acid residues with a reaction handle is a strategy that allows for a site-specific chemical conjugation, leading to strictly controlled DARs. Schultz and co-workers have developed protein expression systems (bacteria, yeast, and mammalian cells) where *p*-acetylphenylalanine containing a carbonyl group is genetically encoded by introducing a unique codon-tRNA synthetase (Axup et al., 2012; Tian et al., 2014) (Fig. 5A). Engineered antibodies containing *p*-acetylphenylalanine residues are produced using either of the expression systems, and the carbonyl groups introduced react with alkoxyamine-functionalized linkers to provide oxime-conjugated ADCs. Other examples are *p*-azidomethyl-_L_-phenylalanine (Zimmerman et al., 2014) and *N*6-((2-azidoethoxy)carbonyl)-_L_-lysine (VanBrunt et al., 2015) (Fig. 5B). The incorporated azide groups are used for conjugation with alkyne-functionalized linkers through the copper-catalyzed Huisgen cycloaddition (called “click chemistry” in general) to provide triazole-linked ADCs. Zimmerman et al. used this method to conjugate monomethyl auristatin F (MMAF) with the trastuzumab, which afforded a potent ADC (Zimmerman et al., 2014). All functional groups in the antibody sequence are tolerant of both conjugation reactions. Thus, DARs can be tightly controlled by adjusting the degree of non-natural amino acid incorporation and fully using the reaction handles incorporated for conjugation. Bioorthogonal conjugation of azide-incorporated antibodies can be achieved by using strained cyclooctyne-functionalized linkers that do not require a cytotoxic, oxidative copper catalyst (Fig. 5C). However, the non-natural amino acid-based methodology generally requires special techniques and biological agents for the genetic engineering process, and the incorporated non-natural amino acid residues could potentially invoke undesired immunological response. Further efforts to solve such issues will make this method truly practical and versatile in industrial production of ADCs.

### Enzymatic conjugation

Several enzymes have been used for conjugating the native or genetically engineered antibody with the payload or for installing unique reaction handles on the antibody scaffold for the following chemical conjugation. These enzymes modify the antibody in a site- or amino acid sequence-specific manner. Furthermore, the reaction sites in native mAbs or handles that are genetically introduced are designed to specifically react with counterpart functional groups. Thus, (chemo)enzymatic approaches generally allow for site-specific conjugation leading to tightly controlled DARs.

#### Transpeptidation using sortase

**Figure 6:**
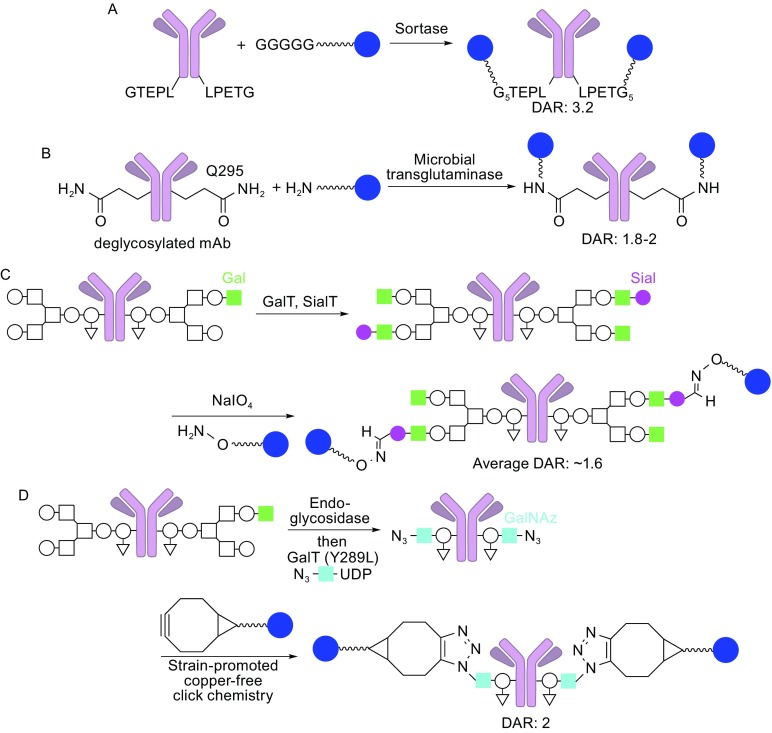
**Site-specific (chemo)enzymatic conjugation**. (A) Sortase-mediated conjugation. Sortase attaches oligoglycine-functionalized linkers to LPETG tags on the mAb. (B) Microbial transglutaminase-mediated conjugation. The enzyme attaches an ADC linker possessing a primary amine to Q295 of the heavy chain (DAR: 1.8–2, high homogeneity). (C) Conjugation using β-1,4-galactosyltransferase (GalT) and α-2,6-sialyltransferase (SialT) (light green square: β-1,4-galactose, magenta circle: sialic acid). The aldehyde groups installed react with alkoxyamine-functionalized linkers (average DAR: ~1.6). (D) GlycoConnect technology using endoglycosidase, galactosyltransferase, and *N*-azidoacetylgalactosamine (GalNAz, light blue square). The azide groups installed react with strained cyclooctyne-functionalized linkers (DAR: 2, high homogeneity)

Sortase A from *Staphylococcus aureus* recognizes the LPXTG (X: any amino acid) motif, cleaves the threonine-glycine (T-G) bond, and attaches an oligoglycine (oligo-G)-containing molecule. Various cargo can be fused to the oligo-G for sortase A-mediated conjugation: peptides, proteins, nucleic acids, and so on (Popp et al., 2009; Witte et al., 2012). For example, Ploegh and co-workers demonstrated stoichiometric and site-specific conjugation of a biotinylated class I MHC-restricted epitope to an antibody against the C-type lectin DEC205 containing a LPETG-His_6_ sequence at its C-terminus of the heavy chain (Swee et al., 2013). The resulting conjugate retained epitope generation ability upon binding to dendritic cells and enabled monitoring of intracellular processes *in vitro* and *in vivo*. Beerli and co-workers demonstrated the potential of this powerful approach for stoichiometric site-specific ADC conjugation (Beerli et al., 2015) (Fig. 6A). They introduced the recognition motif LPETG to the C-termini of the light and heavy chains of various mAbs. Then, the small molecule payload monomethyl auristatin E (MMAE) containing penta-G was conjugated to the mAbs in the presence of sortase A. The resulting conjugates (DAR: approximately 3.2, monomer content: >96%) showed no adverse effect on antibody binding to the counterpart antigens. Further, these conjugates exerted *in-vitro* cell killing activities comparable to the corresponding conjugates generated by traditional ADC conjugation methods, including the FDA-approved ADCs Adcetris^®^ and Kadcyla^®^. This method can also be used for site-specific conjugation of the single-chain variable fragment (scFv) derived from mAbs (Madej et al., 2012).

#### Transpeptidation using microbial transglutaminase

The use of bacterial transglutaminases is a powerful approach for site-specific incorporation of the payload into the antibody (Fig. 6B). A transglutaminase derived from *Streptomyces mobaraensis* catalyzes transpeptidation where a primary amine-containing linker is covalently attached to the primary amide side chain of a specific glutamine (Q295) within deglycosylated antibodies, resulting in ADCs with a defined DAR of 2 (one conjugation site per heavy chain) (Jeger et al., 2010; Dennler et al., 2014). An N297Q mutation prior to this conjugation provides two more reaction sites (DAR = 4). This method is quite advantageous in terms of practical ADC production as the glycosidase and transglutaminase directly modify and conjugate native mAbs with the payload, without the need for genetic engineering. Strop and co-workers developed an alternative version using a peptide sequence-specific transglutaminase (Strop et al., 2013). This enzyme recognizes and utilizes LLQG motif that is genetically incorporated, resulting in site-specific antibody-drug conjugation. Another advantage of this LLQG-specific bacterial transglutaminase is that conjugation sites can be flexibly laid by inserting this short peptide motif within the antibody structure. They demonstrated the potential of this strategy by preparing two ADCs that showed tightly controlled DARs (~1.9) and comparable cytotoxicity and tolerability profiles.

#### N-Glycan engineering

Asn297 (N297) within the Fc domain and the *N*-glycan on this residue are conserved in all IgG classes, making these components attractive reaction sites for broadly applicable ADC conjugation. Zhou and co-workers developed incorporation of an aldehyde group on the *N*-glycan terminus using β-1,4-galactosyltransferase (GalT) and α-2,6-sialyltransferase (SialT) (Fig. 6C)(Zhou et al., 2014). These two enzymes introduce a sialic acid on each *N*-glycan terminus, which is subsequently converted into an aldehyde group using NaIO_4_ under mild oxidation conditions. The aldehyde groups generated are then used to conjugate aminooxy-functionalized payloads. In their study, this conjugation method gave an average DAR of 1.6, which was approximately the same number of sialic acid residues introduced per antibody. Unfortunately, the oxidation step using NaIO_4_ can oxidize methionine residues within the antibody, and DAR distribution is wide due to low conversion. Another approach is to incorporate non-natural saccharides possessing orthogonal reaction handles into the antibody. One of the latest technologies based on this strategy is the GlycoConnect technology developed by van Delft and co-workers (van Geel et al., 2015) (Fig. 6D). The glycan chain at Asn297 was trimmed using the endoglycosidase Endo S2 and then azide groups were introduced using a mutant galactosyl transferase GalT(Y289L) and *N*-azidoacetylgalactosamine (GalNAz). The azide handles were used for a strain-promoted click reaction with payloads, resulting in stable and homogeneous ADCs with tightly controlled DARs (predominant at 2 in most cases). The biggest advantage of this technology is that it gives consistent results regardless of the heterogeneity of the *N*-Glycan forms, meaning that it can be used for any IgG isotypes with various *N*-glycosylation profiles.

### Cleavable linkers

**Figure 7:**
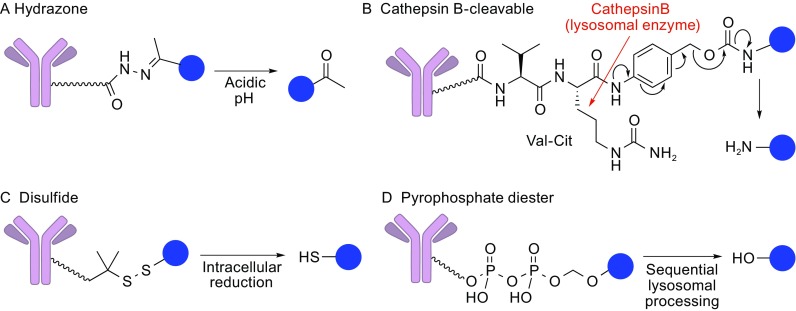
**Cleavable linkers**. (A) Hydrazone linker. This linker is cleaved in the acidic environment (i.e., endosome and lysosome). (B) Cathepsin B-cleavable peptide linker such as valine-citrulline-*p*-aminobenzyloxycarbonyl (PABC) and valine-alanine-PABC. The PABC moiety enables release of free payload molecules in a traceless manner. (C) Disulfide-containing linker. The disulfide bond is reduced by intracellular reducing molecules (e.g., glutathione) to release the payload. (D) Pyrophosphate diester. This stable, hydrophilic linker is cleaved in lysosomes and free payload molecules are released

A major class of ADC linkers is the cleavable linker (Fig. 7). Cleavable linkers are designed to be cleaved by responding to an environmental difference between the extracellular and intracellular environments (pH, redox potential, etc.) or by specific lysosomal enzymes. In most cases, the linkers in this class are designed to release parental payload molecules after bond cleavage. Such traceless drug release mechanisms allow researchers to estimate cytotoxic potency of the conjugated payload based on known pharmacological parameters of the free payload.

#### Hydrazone linker

Hydrazone, an acid-labile group, is used as a cleavable linker that releases free drug through hydrolysis once an ADC is transported to acidic endosomes (pH 5.0–6.0) and lysosomes (pH about 4.8) (Fig. 7A). The chimeric antibody BR96-doxorubicin conjugate (BR96-DOX) was developed with the hydrazone conjugation strategy. BR96-DOX was advanced to a Phase II human clinical trial in metastatic breast cancer (Tolcher et al., 1999). The toxicity profile of the conjugate was considerably improved compared to free doxorubicin administration. However, gastrointestinal toxicity was still prominent and clinical outcomes were not satisfying due to its low tolerability. Another example is the anti-CD33 antibody calicheamicin conjugate, Mylotarg^®^ (Linenberger et al., 2001; Sievers et al., 2001). Mylotarg^®^ showed encouraging clinical results and was approved in 2000. However, as mentioned earlier, it was withdrawn from the market in 2010 due to a lack of clinically significant improvement of patient outcome. Both unsuccessful ADCs suffered from toxicities and low tolerability, which seems to be attributed to lability of the hydrazone linker during circulation. Indeed, ADCs with the hydrazone linker undergo slow hydrolysis under physiological conditions (pH 7.4, 37°C), resulting in a slow release of the toxic payload (Laguzza et al., 1989).

#### Cathepsin B-responsive linker

Cathepsin B is a lysosomal protease that is over-expressed in various cancer cells and involved in numerous oncogenic processes in humans (Gondi and Rao, 2013). Cathepsin B has a relatively broad scope of substrate, but it preferentially recognizes certain sequences such as phenylalanine-lysine (Phe-Lys) and valine-citrulline (Val-Cit) and cleaves a peptide bond on the C-terminal side of such sequences. In particular, Val-Cit and Val-Ala linkers coupled with *p*-aminobenzyloxycarbonyl (Val-Cit-PABC and Val-Ala-PABC) are the most successful cleavable linkers for ADCs (Dubowchik et al., 2002; Hartley, 2011) (Fig. 7B). Upon internalization through endocytosis and transportation to lysosomes, cathepsin B selectively cleaves this linker and cytotoxic payloads are released from the ADC in a traceless manner. The PABC moiety functions as a spacer between Val-Cit moiety and the payload, allowing cathepsin B to exhibit its full protease activity to the linker connected to a bulky payload molecule such as doxorubicin (Dubowchik et al., 2002). This linker was used to construct the chimeric anti-CD30 antibody-MMAE conjugate, or brentuximab vedotin (Adcetris^®^) (Younes et al., 2010).

#### Disulfide linker

Glutathione sensitive linker is another common cleavable linker (Fig. 7C). This strategy relies on the higher concentration of reducing molecules such as glutathione in the cytoplasm (1–10 mmol/L) (Wu et al., 2004) compared to the extracellular environment (about 5 µmol/L in blood) (Mills and Lang, 1996). A disulfide bond is embedded within the linker and resists reductive cleavage in circulation. However, upon internalization, abundant intracellular glutathione reductively cleaves the disulfide bond to release the free payload molecule. To further enhance stability during circulation, methyl groups are often installed next to the disulfide bond (Saito et al., 2003). This class of linker has been employed in Mylotarg^®^ (Sievers et al., 2001), and more recently, in several maytansine-based candidates in clinical trials (Widdison et al., 2015).

#### Pyrophosphate diester linker

Recently, Garbaccio and co-workers developed a novel cleavable linker with a pyrophosphate diester structure (Fig. 7D) (Kern et al., 2016). This anionic linker has greater aqueous solubility than traditional linkers and excellent circulatory stability. Furthermore, upon internalization, the pyrophosphate diester gets promptly cleaved through the endosomal-lysosomal pathway to liberate unmodified payload molecules. The authors speculate that the pyrophosphate diester goes through a two-step enzymatic linker cleavage that releases a payload-monophosphate molecule and then a free payload, although the enzyme(s) involved in this process have not yet been identified. With this encouraging result, they set out to construct conjugates of the anti-human CD70 antibody and various glucocorticoids using this linker. The ADCs constructed showed great stability in human plasma (intact *in vitro* up to 7 days) and fast linker cleavage and release of free payload molecules in lysosomes. Interestingly, each conjugate released a free payload at different rates, depending on the substituent group proximal to the pyrophosphate moiety. This result suggests that the rate of release could be fine-tuned by further structural modifications. In addition, one of the ADCs containing fluticasone propionate exerted remarkable potency (EC_50_: 0.37 nmol/L) in CD70-positive 786-O cells, comparable to free fluticasone propionate (EC_50_: 0.25 nmol/L). These results demonstrate the potential of the pyrophosphate diester linker for the future development of therapeutically practical ADCs.

### Non-cleavable Linkers

**Figure 8:**
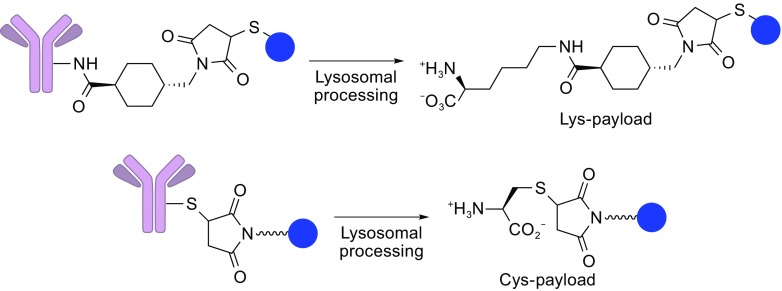
**Non-cleavable linker**. The chemical stability of the non-cleavable linker withstands proteolytic degradation. Cytosolic/lysosomal degradation of the mAb moiety liberates the payload molecule linked to an amino acid residue derived from the degraded mAb

Non-cleavable linkers consist of stable bonds that are resistant to proteolytic degradation, ensuring greater stability than that of cleavable linkers. Non-cleavable linkers rely on complete degradation of the antibody component of ADC by cytosolic and lysosomal proteases, which eventually liberates a payload molecule linked to an amino acid residue derived from the degraded antibody (Fig. 8). As such, when coupled with a non-cleavable linker, the payload structure must be carefully selected and designed so that payload can exert comparable or even better anti-tumor potency in such a modified form. For that purpose, it may be necessary to examine PK/PD and toxicity profiles of all possible metabolites of ADCs with non-cleavable linkers. A successful example of ADCs using a non-cleavable linker is the humanized anti-HER2 antibody-maytansine conjugate trastuzumab emtansine (T-DM1, or Kadcyla^®^) (LoRusso et al., 2011; Verma et al., 2012).

## CONCLUDING REMARKS AND OUTLOOK

In this article, we have reviewed the concept and clinical potential of ADCs and various conjugation/linker strategies for constructing this new class of molecules (Table 1). Compared to traditional small molecule-based chemotherapy, well-designed ADCs have several distinct features and clinical advantages, including preferable PK/PD and biodistribution (which are generally similar to that of native IgGs), broader therapeutic window, and flexibility of molecular customization. As exemplified in the successes of the FDA approved Adcetris^®^ and Kadcyla^®^, this new therapeutic modality has huge potential for anti-cancer therapy and has attracted a great deal of attention from researchers and clinicians. Indeed, significant advances have been made in ADC technologies, with about 60 ADCs currently in clinical trials. This emerging molecular platform is expected to become mainstream in anti-cancer therapeutics in the near future. Despite its potential, further understanding biochemical, immunological, pharmacological, and molecular aspects of ADCs must be pursued to better design and develop effective ADCs. While choice of target antigens and payloads is important, antibody-payload conjugation methods and linker chemistry are also crucial elements for producing successful ADCs. In particular, instability of the linker and heterogeneity of the product (i.e., broad distribution of DARs) often negatively impacts ADC efficacy and therapeutic window, which often leads to difficulty or limitation in the optimization for clinical application and eventual failure in clinical trials. To overcome these problems, current efforts are directed toward developing novel stable linkers (with or without a payload release mechanism) and site-specific conjugation methods enabling construction of homogeneous ADCs. Further investigations along this line will provide greater insights and sophisticated strategies from medicinal chemistry and pharmacology standpoints, leading to innovative cancer therapeutics in the future.

**Table 1:** Advantages and disadvantages of the conjugation and linker chemistries described

	Strategy	DAR^a^	Advantages	Disadvantages
Chemical conjugation	Lysine coupling	0–7	Simple process Used in FDA-approved and clinically tested ADCs	Distributed DAR Heterogeneous mixtures of products Potential reduction of antigen binding
	Cysteine coupling	0, 2, 4, 6, 8	Simple process Used in FDA-approved and clinically tested ADCs	Heterogeneous mixtures of products Increased clearance rate with high DAR
	THIOMAB	2	Defined DAR Homogeneity	Requires genetic engineering
	Cysteine rebridging	4	Defined DAR Homogeneity High structural stability	Potential disulfide scrambling
	Non-natural amino acid	2	Defined DAR Homogeneity	Requires special techniques and biological agents Potential immunogenicity
	Sortase	3–4	Tightly-controlled DAR No adverse effect on antibody binding	Requires incorporation of LPETG motif on the heavy chain
(Chemo) enzymatic conjugation	Microbial transglutaminase	2	Defined DARs Homogeneity	Requires removal of *N*-glycan on N297
	Glycan engineering (GlycoConnect)	2	Defined DARs Homogeneity	Requires multiple steps (i.e., *N*-glycan trimming, glycosylation, and conjugation)
	Hydrazone		pH-responsive cleavage	Premature cleavage during circulation
	Val-Cit^b^-PABC^c^,		Stability during circulation	Hydrophobicity
	Val-Ala-PABC^c^		Traceless release of payload	
Cleavable Linker	Disulfide		Intracellular release of payload	Potential premature cleavage during circulation
	Pyrophosphate diester		Stability during circulation Hydrophilicity Traceless release of payload	Unknown mechanism of lysosomal cleavage
Non-cleavable Linker	Stable linker without cleavage mechanism		Stability during circulation	An amino acid residue attached on the released payload

^a^DAR, Drug-to-antibody ratio; ^b^ Cit, citrulline; ^c^ PABC, *p*-aminobenzyloxycarbonyl
